# Additively Combining Utilities and Beliefs: Research Gaps and Algorithmic Developments

**DOI:** 10.3389/fnins.2021.704728

**Published:** 2021-10-01

**Authors:** Anush Ghambaryan, Boris Gutkin, Vasily Klucharev, Etienne Koechlin

**Affiliations:** ^1^Centre for Cognition and Decision Making, HSE University, Moscow, Russia; ^2^Ecole Normale Supérieure, PSL Research University, Paris, France

**Keywords:** additive strategy, uncertain and volatile environment, normalized utility, state belief, value-based decision making, one-armed bandit task, MIX model

## Abstract

Value-based decision making in complex environments, such as those with uncertain and volatile mapping of reward probabilities onto options, may engender computational strategies that are not necessarily optimal in terms of normative frameworks but may ensure effective learning and behavioral flexibility in conditions of limited neural computational resources. In this article, we review a suboptimal strategy – additively combining reward magnitude and reward probability attributes of options for value-based decision making. In addition, we present computational intricacies of a recently developed model (named MIX model) representing an algorithmic implementation of the additive strategy in sequential decision-making with two options. We also discuss its opportunities; and conceptual, inferential, and generalization issues. Furthermore, we suggest future studies that will reveal the potential and serve the further development of the MIX model as a general model of value-based choice making.

## Introduction

**FIGURE 1:**
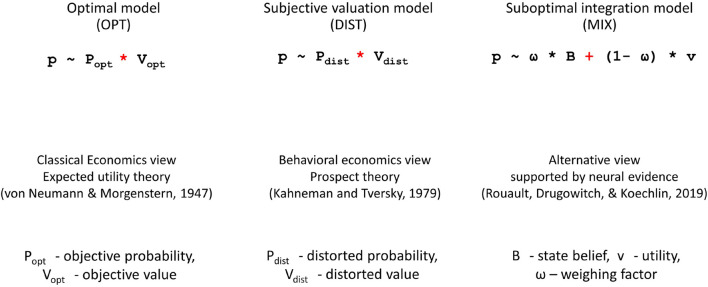
Probability of choosing an option according to classical economics view **(the leftmost panel)**, behavioral economics view **(the middle panel)**, and a recently developed MIX model **(the rightmost panel)**.

A fundamental assumption in classical economics is that reward magnitudes and reward probabilities (computational components), following the expected utility theory (von Neumann and Morgenstern, 1947), are integrated in optimal way, that is, *multiplicatively*, for deriving option values and making choices. To explain systematic violations of optimal decision making in humans, behavioral economists have developed the prospect theory (Kahneman and Tversky, 1979). According to this theory, humans perform optimal combination (integration) of computational components, as described in the expected utility theory, but make computations operating on distorted representations of rewards and their probabilities (subjective valuation). This approach cannot dissociate the sub-optimality of the computation and the distortion of computational components since the suboptimality can be hidden behind variations in distortions. Hence, the prospect theory may lead to models that fit human choices but are not indicative of underlying computational mechanisms. This tends to undermine the core aim of behavioral economics, that is, to understand human behavior *per se*, and limit the potential of model-based studies of neural mechanisms (Love, 2015). A recent study (Rouault et al., 2019) hypothesized an alternative view, that is, the *additive strategy* of option value derivation (MIX model) and contrasted it with both the *multiplicative strategy* (OPT model) and the *subjective valuation* (DIST model). The key aspects of the models are shown in Figure 1.

According to the OPT model, the probability *p* of choosing an option varies with the expected value of the choice option based on multiplying its reward magnitude and reward probability (leftmost panel of Figure 1). Following the conventional reinforcement learning (RL) framework, we assume agents observe options available in the environment; make a choice acting in the instructed way for that; and receive reward. The latter is both the reinforcer and the feedback information about the environment (the reward hypothesis). This means we focused on how the reinforcer guides behavior but not on what exact utilities are obtained from the options (Juechems and Summerfield, 2019). That is why, we consider the optimal combination of reward magnitude and reward probability attributes as a variable guiding behavior in the OPT model. The DIST model assumes that the probability *p* of choosing an option varies with the *subjective* expected value of the choice option based on multiplying the subjective estimates of the reward magnitude and reward probability (middle panel of Figure 1). Whereas the MIX model hypothesizes that the probability *p* of choosing an option varies proportional to the *linear combination* of the state belief (rationally derived mapping of reward probabilities onto choice options) and the normalized utility associated with the choice option. Parameter *ω* weighs the contribution of the state belief against the normalized utility to the choice probability *p* (rightmost panel of Figure 1). Hence, *ω* can be viewed as an analog of the risk-aversion measure in the expected utility framework. Relatively high values of parameter ω favor high reliance on state beliefs compared to utility information, thus yielding relatively safe choices, whereas the opposite indicates risk-seeking choices. Both state beliefs and utilities of choice options are derived through a multi-step computational algorithm, which as it was shown by Rouault et al. (2019) represent a mechanistic neurocomputational account of human choices in an uncertain and volatile environment of value-based decision making.

In the next section, we review behavioral and neural findings supporting the additive strategy hypothesis of value-based decision making and the rationale behind the additive strategy. To make clear the reasoning behind choices, we next present the algorithmic implementation of the MIX model as well as those of the DIST and OPT models. Conceptual connections of the free parameters of the models are explained. Afterward, we discuss research questions and opportunities in light of the sub-optimal computational strategy of additively combining state beliefs and normalized utilities; and briefly address conceptual and inferential issues arising from the shift of the framework of human behavior estimation from the subjective valuation to the additive strategy. In the final section, we specify main directions for further development of the MIX model which would allow us to generalize it to more complex and real-life environments of decision making.

## Additive Strategy Fitting Human Choices

**FIGURE 2:**
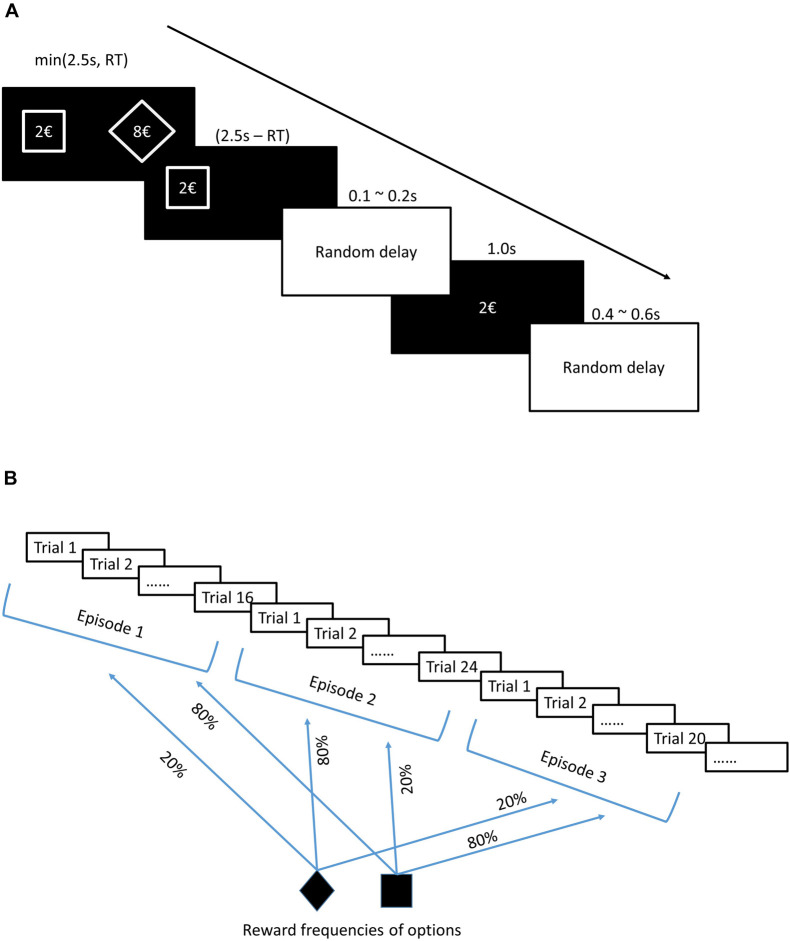
**(A)** Trial structure. In each trial, subjects see two forms (options), a diamond and a square, each proposing a reward in euros randomly chosen from the set {2, 4, 6, 8, and 10}. After making a choice, subjects only see the chosen option on the screen, followed by a display of the outcome of the choice in the center of the screen. The average duration of a trial was 4.15 s. After displaying two available options on the screen, subjects were given 1.5 s for thinking and responding by pressing one of two instructed buttons on the keyboard, left button for choosing the option on the left side of the screen and right button for choosing the option on the right side of the screen. The outcome of the trial was displayed 1.0 s. The delay of the outcome display was 0.1–0.2 s. The inter-trial delay was 0.4–0.6 s. **(B)** Experimental design. The outcome could be zero or equal to the proposed reward (shown on the first screen of each trial) with some probability that subjects were not informed about. However, they could derive the reward frequencies through experience. By experimental design, 20 and 80% reward frequencies were assigned to two options and switched between them after a random number of trials (16, 20, 24, or 28). Subjects were not informed about switches but could detect them throughout the experiment based on feedbacks (outcomes). Each subject went through 19 switches of reward frequencies, which divided the task into 20 episodes (a series of trials within which no change of reward frequencies occurs).

Rouault et al. (2019) used a sequential decision-making task (Dayan and Daw, 2008), where subjects were not provided with information about reward probabilities but could learn them over trials. Subjects also had to adjust their choices as the reward probabilities switched between options over a random number of trials. Thus, the authors used a variation of the one-armed bandit task (hereafter, MIX task), which recreated an uncertain (reward frequencies were not known to subjects) and volatile (switches were not signaled to subjects) environment of decision making (unstable uncertainty). The structure of a typical trial is presented in Figure 2A and the implementation of uncertainty and volatility is schematically explained in Figure 2B

Rouault et al. (2019) found that the MIX model (reflecting the additive strategy) accounted for human choices better than the OPT and DIST models did although choice simulations of the DIST model were quite close to human choices (subjects acted *as if* they followed the subjective valuation model). The study also showed the neural signature of the MIX model. The brain region that exhibited activations varying with decision entropy according to the MIX model was the dorsomedial prefrontal cortex (dmPFC). Besides, its activation decreased when state beliefs or normalized utilities associated with chosen options increased, with no interactions between these two effects. These neural findings are strong evidence that the dmPFC guides choices and computes them through combining normalized utilities and state beliefs additively as proposed by the MIX model.^1^ On the other hand, no neural evidence was observed to support the notion of subjective probabilities assumed in the DIST model. Taken together, these findings provide significant evidence that behavioral models of expected utility with multiple distortions of computational components may fit human choices but they do not necessarily reflect the reasoning behind (neural and computational mechanism of deriving and making choices). In several previous studies with various value-based decision-making tasks (Kolling et al., 2014; Scholl et al., 2015) or various protocols of the MIX task (Donahue and Lee, 2015) researchers have also noticed that humans’ and non-human primates’ choices are better explained by models which derive option values as linear rather than optimal combinations of reward magnitude and reward probability attributes. Furthermore, a recent study (Farashahi et al., 2019) which utilized an experimental paradigm close to the MIX task and was conducted with primates both human and non-human reported findings largely coinciding with and complementing conclusions by Rouault et al. (2019).

In a complementary study, Farashahi et al. (2019) used a gambling task (requiring decision making under risk), a mixed learning task (requiring decision making under stable or volatile uncertainty), and probabilistic reversal learning tasks (PRL, requiring decision making under low or high volatility). The authors proposed a hybrid model that comprises both additive and multiplicative strategies, with a free parameter weighing their relative contribution to choices. Accordingly, parameter estimation allowed the authors to measure the relative contribution of the additive and multiplicative strategies; and within the additive strategy, to measure the relative contribution of reward magnitudes and reward probabilities to choices. Farashahi et al. (2019) found that both monkeys and humans predominately adopt a multiplicative strategy under risk (reward probabilities are provided explicitly). And both switch to an additive strategy under uncertainty (reward probabilities are not provided explicitly and should be learned). Moreover, Farashahi et al. (2019) found that within the additive strategy, both humans and monkeys increasingly rely on reward magnitudes relative to probabilities as the environment changes from stable uncertainty to low volatility and from low to high volatility. Hence, the volatility level appears to affect the level of reliance on reward magnitudes relative to probabilities, whereas the multiplicative relative to additive strategy is used according to the availability of probability information: if explicitly provided, both humans and monkeys appear to predominantly use the multiplicative strategy; otherwise, the additive strategy is adopted regardless of whether the uncertain environment is stable or volatile. Farashahi et al. (2019) also found that the changes in the difference between reward magnitudes of options were associated with changes in the activation of dorsolateral prefrontal cortex. Importantly, the strength of the association increased with the increase of the behavioral weighting of the reward magnitude relative to the reward probability under high volatility. So, the change of the neural signal associated with the difference of options with respect to their reward magnitudes is accompanied by the change of subjects’ reliance on that information. Consistent neural findings are reported by a previous study with PRL task in non-human primates (Donahue and Lee, 2015; Massi et al., 2018). How findings by Farashahi et al. (2019) and Rouault et al. (2019) are related is discussed next.

The results of the two studies coincide in finding that humans adopt an additive computational strategy when making choices under uncertainty. The additive strategy was dominant under uncertainty not only in condition of stable reward frequencies but also in conditions of low and high volatility of reward frequencies, as reported by Farashahi et al. (2019). Furthermore, Farashahi et al. (2019) provides rich evidence of variations in human behavior by manipulating the level of volatility and by manipulating the environmental uncertainty vs. riskiness. The hybrid model applied by Farashahi et al. (2019) is a composition of hierarchically nested computational strategies and effectively captured behavioral switches between those strategies without revealing the neurocomputational mechanism of the switches but allowing for linear and non-linear transformations of computational components. Whereas Rouault et al. (2019) proposes a mechanistic account – a multi-step computational algorithm of deriving computational components upon which the additive strategy is implemented. As such, the MIX model extends the evidence in support of the additive strategy to the reasoning behind, specifically, processing of incomplete information from the environment into choice making; learning the environment; and adapting to changes in it. A key step of the MIX algorithm is the normalization of utilities, which makes them commensurable to state beliefs like probabilistic variables (see the justification of the step in section “Algorithmic Implementation of the Additive Strategy”) and mitigates the influence of very large rewards with very low probability. Importantly, the OPT model (the optimal combination) with normalized utilities and state beliefs (rationally derived under uncertainty) is equivalent to the sum of normalized utilities and state beliefs with a specific value of the weight parameter ω in the MIX model. And since Rouault et al. (2019) found that the average estimate of the parameter ω was significantly higher than its critical level whereby the MIX model reduces to the OPT model with utilities normalized across choice options, they proposed the MIX model is a general model that encompasses the OPT model as a special case. This ensures the MIX algorithm has the capability of detecting behaviors resorting to the OPT model, hence it is also not devoid of flexibility of the composite design of the hybrid model by Farashahi et al. (2019). However, the MIX model was tested and contrasted with classical economics and behavioral economics views, both behaviorally and neuraly, only at one volatility level of an uncertain environment. Probed in various decision-making contexts [environments which are known to give rise to behavioral variations formulated as cognitive phenomena (particularly, loss aversion, reference-dependency) underlying the suboptimality according to the prospect theory (the subjective valuation model)] and supported by neural evidence, the MIX model purports to be the general neurocomputational model of value-based decision-making.

Several other studies have provided empirical evidence in support of the additive model as opposed to the multiplicative model in fitting human and non-human primates’ choices in PRL tasks and enriched evidence of behavioral variations in light of the additive strategy while varying the level of uncertainty (closeness of reward frequencies of two options) and environmental volatility (frequency of switches of reward probabilities between two options). Particularly, Blain and Rutledge (2020) found that a parameter weighting the contribution of reward probability information against the reward magnitude information decreased as the environmental volatility increased. Indirect evidence in support of this is the finding that the increase of environmental volatility is accompanied by the increase of the RL rate (Behrens et al., 2007; McGuire et al., 2014; Blain and Rutledge, 2020). So, subjects speed up the learning as the acquired mappings expire quickly. This is exactly what was found by Farashahi et al. (2019); follows the reasoning by Findling et al. (2021) and is designed to emerge through the MIX model. Specifically, the MIX algorithm rationally updates beliefs about reward probabilities of options as the environment unfolds within trials (see the first and second update of state beliefs in the MIX algorithm in the next section) and across trials (see the third update of state beliefs according to the MIX algorithm). And, the MIX algorithm assigns a measure of reliance on those rationally derived beliefs. Conceptually, the state belief update in the MIX model resembles a combination of evidence-triggered updating and change point estimation of environmental volatility (Gallistel et al., 2014). Alternatively, another study (Farashahi et al., 2017) which also confirmed the additive strategy of reward magnitude and reward probability combination in a protocol close to the MIX task for non-human primates (Donahue and Lee, 2015), proposed a hierarchical structure between the learning of the environmental volatility and the update of option values. So, the additive model manifests as the strategy of option value derivation regardless of the state belief derivation model. Overall, the evidence in support of the additive computational strategy raises concerns regarding conclusions of behavioral and neural studies of decision making, which imply but do not explicitly check whether human choices follow variations in the expected utility (Holt and Laury, 2002; Christopoulos et al., 2009; Glimcher and Fehr, 2014; Blankenstein et al., 2017; Blankenstein and van Duijvenvoorde, 2019). Hence, further studies are needed that would allow us to rethink the value-based decision making. In the upcoming sections, we discuss issues and opportunities for such studies. Before that let us review the rationale behind the additive strategy hypothesis.

The additive strategy hypothesis claims reward magnitude and reward probability attributes independently contribute to choices. Particularly, accordingly to the MIX model, choices are guided by state beliefs (choose the most frequently rewarding option), whereas normalized utilities act as additional appetitive values of choice options [based on an efficient coding mechanism of context-dependent value normalization (Carandini and Heeger, 2012; Louie et al., 2013)]. As such, the additive strategy hypothesis doubts the complex cross-product process of optimal integration of reward magnitudes and reward probabilities under uncertainty. As claimed by Farashahi et al. (2019), the latter results in an integrated value difficult for revisions, whereas derivation and multiple updates of states beliefs are required. The additive strategy implies to separately compare options in each of the two dimensions and flexibly adjust the reliance on the reward probability attribute (the attribute under uncertainty) relative to the reliance on the reward magnitude attribute. So, it is advantageous for learning and choice making under uncertainty. This interpretation by Farashahi et al. (2019) is consistent with the finding by Rouault et al. (2019) that the parameter ω weighting the reliance on the probability attribute vs. magnitude attribute was not 0.5 (mean estimate was 0.69, and the standard error of mean was 0.06 across subjects). And the reliance on reward probabilities decreased with the increase of the environmental volatility as reported by Farashahi et al. (2019). So, subjects predominantly relied on the reward probability attribute (the derived belief about them) under uncertainty (an indication of risk-aversion) and reduced that tendency as the derived state beliefs were discredited by the increased environment volatility. Finally, as noticed by Koechlin (2020), an experimental condition when subjects are explicitly instructed about reward probabilities corresponds to a hyper-volatile situation (each trial is unrelated to the preceding ones). And in such condition, choices confirming the subjective value hypothesis still adhere to an additive strategy with equal reliance on reward probability and reward magnitude attributes. So, the additive strategy is a heuristic, an adaptive behavior in uncertain and volatile environments which endows with efficiency in inferring external contingencies and flexibility in making choices.

## Algorithmic Implementation of the Additive Strategy

**FIGURE 3:**
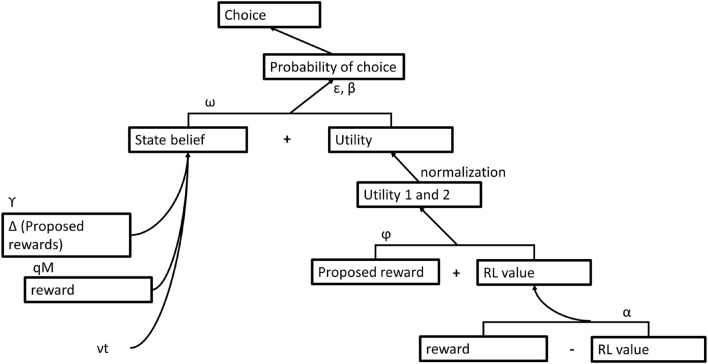
Scheme of the computational algorithm of the MIX model.

The computational algorithm of the MIX model is schematically presented in Figure 3. According to the MIX model, the probability (_*p*_) of choosing an option varies with its state belief^2^ (_*B*_), whereas the option’s utility (_*v*_) additively contributes to that choice probability:


$$p∼\omega^{*}B+{\left(1-\omega \right)}^{*}\nu ,$$

where, parameter ω is the relative reliance on state belief information.

After the derivation of choice probabilities, the MIX algorithm assumes soft-max rule of option selection with inverse temperature β (Acerbi et al., 2014) and lapse rate ε in order to incorporate the tendency of occasionally choosing a low value option (as exploration or incorrect button pressing).


$$p_{i}={\left(1-\varepsilon \right)}^{*}\frac{\exp\left(\beta^{*}\left(w^{*}B_{i}+{\left(1-w\right)}^{*}v_{i}\right)\right)}{\sum_{i=1,2}\exp\left(\beta^{*}\left(w^{*}B_{i}+{\left(1-w\right)}^{*}v_{i}\right)\right)}+\frac{\varepsilon }{2},$$

where, *i* = 1,2. From here, we can derive that the decision variable relating choice probabilities of the available options, the log-odds of two options, _$\text{log}\frac{p_{1}}{p_{2}}$_, varies with the sum of the difference between utilities (_*v*_1__ and _*v*_2__) and the difference between state beliefs (_*B*_1__ and _*B*_2__) of two options assuming _ε<<1_.


$$\log\frac{p_{1}}{p_{2}}∼\omega^{*}\left(B_{1}-B_{2}\right)+{\left(1-\omega \right)}^{*}\left(v_{1}-v_{2}\right)$$

The MIX model incorporates option value learning into the computational algorithm rather than presumes the proposed rewards (OPT model) or the distorted rewards (DIST model) as values. Specifically, the learning passes through observing proposed rewards of choice options; adjusting values of choice options according to feedbacks (outcomes of choices) *via* RL; deriving utilities of choice options as a linear combination of proposed rewards and RL-values; and finally, normalizing utilities of choice options. At the choice between options in a trial, utilities of choice options are weighted averages of the RL-values (ν^*RL*^) and proposed rewards (ν^*PR*^):


$$v_{i}=\frac{\varphi^{*}v_{i}^{PR}+{\left(1-\varphi \right)}^{*}v_{i}^{RL}}{\underset{i}{\Sigma }\left(\varphi^{*}v_{i}^{PR}+{\left(1-\varphi \right)}^{*}v_{i}^{RL}\right)},$$

where, parameter φ is the reliance on the proposed rewards relative to the RL-values (weighs proposed rewards in the current trial against learned values up to the current trial), and *i* = 1, 2 denoting choice options. The RL-value of the chosen option is updated according to the Rescorla–Wagner rule (Rescorla and Wagner, 1972) with learning rate parameter α as follows:


$$\nu^{RL}\leftarrow \nu^{RL}+\alpha^{*}\left(reward-\nu^{RL}\right).$$

Importantly, the MIX model assumes divisive normalization of utilities of the available options before integrating them into the decision variable computation. Rouault et al. (2019) suggested that the context-dependent divisive normalization mechanism (Louie et al., 2015) could underlie the distortion of monetary rewards hypothesized in the subjective valuation. As previously observed, the normalization is applied to various problems across many brain regions, modalities, and systems (Carandini and Heeger, 2012). Particularly, higher-order cortical areas involved in valuation signaling demonstrate spatial context-dependence: the neural signal of an option value depends not only on the value of that option but also on the value of alternative options (Louie et al., 2013). Removing the divisive normalization over utilities in the model MIX degraded the fit as reported by Rouault et al. (2019), which confirms the normalization as a step in the computational algorithm of learning and choice making. Similarly, the reduced variations of the model MIX, particularly, pure RL and no RL compositions of utilities were inferior to the full MIX algorithm in their fitting human choices. Importantly, Rouault et al. (2019) found that the dmPFC which encoded variations in the decision variable (computes choices) exhibited activations associated with normalized utilities but not with its value components, the proposed rewards and the RL-values. Combining this finding with the linear association of changes in the proposed rewards (a prospective value) and the RL-values (a retrospective value) with changes in the activations of vmPFC and lateral orbitofrontal cortex, respectively, Rouault et al. (2019) concluded linear combination of two reward-related variables (functionally distinct components of an option value) into normalized utilities of choice options.

State beliefs are inferred as they would be inferred by a rational agent (OPT model). Specifically, under uncertainty state beliefs are updated as the subject gets new information from the environment: first, when subjects observe the proposed rewards:


$$B_{i}\leftarrow e^{\gamma *\left(v_{i}^{RP}-v_{\left(3-i\right)}^{PR}\right)}*B_{i},$$

where, *i* = 1, 2, and parameter γ is the interdependence bias between reward frequency and reward magnitude;

second, when subjects see the outcome of their choice:


$$B_{1}\leftarrow q^{I*}{\left(1-q\right)}^{\left(1-I\right)*}B_{1}andB_{2}\leftarrow {\left(1-q\right)}^{I*}q^{\left(1-I\right)*}B_{2},$$

where, *I* is the binarized outcome (zero or non-zero outcome), and parameter _*q*_ is the reward frequency of the best option (the one with the highest reward frequency);

third, at the completion of a trial according to environmental volatility, the probability of switching reward frequencies between two successive trials (Behrens et al., 2007):


$$B_{1}\leftarrow {\left(1-vt\right)}^{*}B_{1}+vt^{*}B_{2}andB_{2}\leftarrow vt^{*}B_{1}+{\left(1-vt\right)}^{*}B_{2},$$

where, parameter _*vt*_ is the environmental volatility. Free parameters of the MIX model are summarized in Table 1.

**TABLE 1:** Free parameters of alternative models.

MIX model	OPT model	DIST model
Environmental volatility, νt	Environmental volatility, νt	Environmental volatility, νt
RL rate, α		
Parameter weighing the proposed reward against the RL-value,φ		
Bias of reward frequency depending on the proposed reward of the option, γ	Bias of reward frequency depending on the proposed reward of the option, γ	Bias of reward frequency depending on the proposed reward of the option, γ
Parameter weighing the state belief against the normalized utility of the option, ω		
Lapse rate, ε	Lapse rate, ε	Lapse rate, ε
Inverse temperature, β	Inverse temperature, β	Inverse temperature, β
Reward frequency of the best option, q	Reward frequency of the best option, q	Reward frequency of the best option, q
		S-shaped or inverted S-shaped distortion of probability, η_*p*_
		S-shaped or inverted S-shaped distortion of reward, η_*r*_
		Convex or concave distortion of probability, x_0p_
		Convex or concave distortion of reward, x_0r_

In the recreated volatile and changing environment of the MIX task, a rational agent would follow the OPT model where reward probabilities (state beliefs) are learned through rational updates according to evolving information in the feedbacks upon choices. The expected utilities (EU) in the OPT model are derived by integrating reward probabilities and values:


$$EU_{i}=V_{i}^{*}B_{i},$$

where, V and B denote proposed rewards and state beliefs, respectively, and i = 1, 2.

In a subjective valuation model (model DIST) the choice derivation repeats that in the OPT model except that the computations are based on distorted probabilities and distorted values. The distortions are implemented using a distortion function by Zhang and Maloney (2012),


$$\log\frac{\tilde{x}}{1-\tilde{x}}=\eta^{*}\log\frac{x}{1-x}+{\left(1-\eta \right)}^{*}\log\frac{x_{0}}{1-x_{0}},$$

where, _*x*_ is the distorted value of a variable _*x*_ (probability or value); parameters _η>0_ and x_0_ (_0<x_0_ <1_) specify distortions: _η>1_ specifies S-shaped distortion, _η<1_ specifies inverted S-shaped distortion, _η=1_ means no distortion; _*x*_0→0__ and _*x*_0→1__ specify convex and concave distortions, respectively.

The total number of free parameters of MIX, OPT, and DIST models are 8, 5, and 9, respectively. This means the models MIX and DIST are comparable in their complexity of explaining systematic deviation of subjects from the optimal model. Rouault et al. (2019) maximized model likelihoods when estimating free parameters of models and selected the best model in fitting human choices according to the Bayesian Inference Criterion.

Inferences about behavior with the MIX model revolve around the following parameters of the model: *vt*α, φ *and* ω. Putting them in a common algorithmic interdependence, the MIX model turns into a tool for studying variations in decision-making mechanisms. The RL rate α, which is an estimate of the options’ value update according to new outcome observations, has previously been shown to be affected by the level of environmental volatility (Behrens et al., 2007; Blain and Rutledge, 2020). Parameter φ indicates how much one relies on a learned value of an option when facing a next proposed reward from that option. So, both α and φ may be affected by the environmental volatility: α – retrospectively when relating current factual outcome observations to those in the past, and φ – prospectively when relating proposed rewards to what has been learned up to the present trial. Furthermore, treating volatility *vt* as a free parameter, the MIX model allows explicitly estimating subjects’ inference of the environmental volatility. Eventually, parameter ω (more precisely, its complement, one – ω) indicates to what extent one considers the option utilities derived *via* the chain of computations described above. If a person predominantly relies on state beliefs or normalized utilities, a tendency analogous to risk aversion or risk-seeking will be suggested, respectively.

Manipulating environmental settings (magnitude and frequency of rewards; levels of uncertainty and volatility of reward magnitude and reward frequency attributes of options; gain versus loss representation of rewards; task goals for subjects, etc.), one can induce context-dependent changes in choice preferences of subjects and associate them with variations in model parameters. Inferences about behavioral changes and underlying mechanisms can then be made (a) based on variations in the strength and directionality of correlations between parameters across experimental conditions; (b) based on variations in model parameters’ configuration within subjects with similar behavior according to model-independent measures; and (c) by comparing sensitivity to changes in reward magnitude and/or probability attributes of options, especially in trials where they suggest opposite preferences. The latter may reveal non-linear thresholding mechanisms underlying the preferential weighting of reward magnitude and probability attributes of options (Koechlin, 2020). Using these experimental and analysis opportunities of the MIX task and algorithm, one can undertake studies for deeper understanding of behavioral variations and further develop the MIX model as an analytical tool for value-based decision making.

## Future Studies. Conceptual and Inferential Issues

The MIX model, representing a multi-step computational algorithm of the additive strategy was tested in an uncertain and volatile environmental which was close to the low volatility condition of experiments by Farashahi et al. (2019). A follow-up study is required where the MIX task is performed in conditions of low and high volatilities as well as in conditions of low and high uncertainty. The aim is to check whether the results are consistent with the behavioral variations reported by Farashahi et al. (2019) and others (Farashahi et al., 2017; Massi et al., 2018; Blain and Rutledge, 2020) and find out which MIX parameters capture those variations with the perspective to associate them with activation variations in the prefrontal cortex.

Despite the consensus and even the theorizing of the divisive normalization (Steverson et al., 2019), we suggest an experimental design with small and large reward magnitudes would allow explicitly checking the utility normalization step of the MIX algorithm and importantly, consider the impact of stake size on uncertainty aversion both in gain and loss domains of reward representation (Bouchouicha et al., 2017); check the reward magnitude’s impact on learning (Wu et al., 2017) and rethink interindividual variability of risk aversion (Fehr-Duda et al., 2009; Kolling et al., 2014; Harrison et al., 2017). The results of these studies will outline the plausibility of the MIX model algorithm and will lay the groundwork for future studies with the aims to integrate the MIX model in the decision theory as a general analytical model.

As for discovering the potential of the MIX model in capturing behavioral variations, studies with a gain-versus-loss representation of rewards, studies with and without a target cumulative gain are suggested. With the application of the MIX model these studies will enrich the current understanding of the risk-aversion variability, also the loss aversion, reference dependence, and diminishing sensitivity–cognitive phenomena constituting in the variability and suboptimality of human choices according to the prospect theory and replicated in further studies (Kahneman and Tversky, 1979; Halevy, 2007; Johnson and Busemeyer, 2010; Ruggeri et al., 2020), from the perspective of underlying computational mechanisms (the reasoning behind). Moreover, designed as decision making under risk or uncertainty, the suggested studies will allow distinguishing computational and neural mechanisms of behavior under the two settings (Tobler et al., 2007). Once the MIX model is confirmed to effectively account for the behavioral variations in response to environmental changes, it can underlie further model-based analysis of neural data and draw a general neural computational mechanism of the choice variability and suboptimality in the prefrontal cortex as advancement of what has been found by Rouault et al. (2019) and others (Tobler et al., 2009; Kolling et al., 2014; Chen and Stuphorn, 2018; Massi et al., 2018; Soltani and Izquierdo, 2019; Jezzini et al., 2021; Trudel et al., 2021).

### Conceptual and Inferential Issues

**FIGURE 4:**
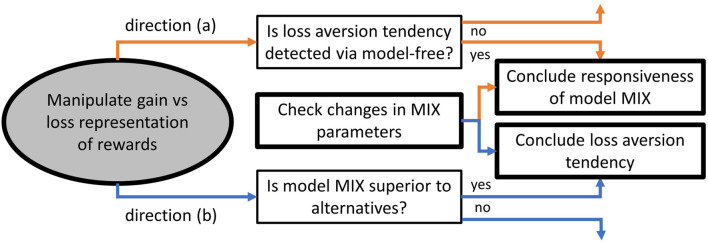
Research directions for the loss aversion as an exemplar behavioral variation compared with choices when outcomes are presented in gain domain. Directions (a) and (b) are presented in orange and blue, respectively.

We suggest two inferential directions for future studies to follow: (a) test the MIX model for responsiveness to behavioral variations, and (b) test for the presence of certain behavioral variations in decision making under uncertainty with the application of the MIX model. The two directions are tightly interrelated and can be designed into the same study. However, they emanate from opposite premises, and the following inferential issue should be considered. Direction (a) presumes that certain behavioral variations are present in decision making under uncertainty. Hence, the MIX model is tested on the ability to detect them *via* its free parameters. By contrast, direction (b) presumes that the MIX model is the appropriate model of behavior under uncertainty. Hence, it can be used for detecting behavioral variations through variations in its free parameter estimates. The abovementioned is an issue of controlling confounding effects. Utilizing model-independent measures of behavioral variations in direction (a) (orange in Figure 4) and determining the best model among alternatives in direction (b) (blue in Figure 4) may resolve the issue. Model-independent measures will justify the search for corresponding evidence in the MIX parameters [direction (a)]. The better fitting to human behavior by the MIX model compared to alternative models will justify the search for behavioral variations in the MIX parameters [direction (b)].

Possible model-independent measures are: (1) variations in learning dynamics (curves) and (2) significance of reward frequencies and proposed rewards for a choice *via* regression analysis. The former will reveal general variations in behavior (number of trials before reaching learning curve plateaus, correct choice proportion at learning curve plateaus), and the latter will suggest the reliance on the reward magnitude attribute relative to the reward probability attribute of options. Importantly, the analysis *via* model-independent measures is limited for two main reasons. First, the abovementioned measures are not specific to a specific behavioral variation; rather, they are general indicators of behavioral variations. Second, the use of measures from normative frameworks of human behavior is related to the following conceptual issue. If humans follow the MIX algorithm for learning and action selection, they are not supposed to reason in terms of EU or subjective values that are inexorably linked to multiplicative integration strategies. Alternatively, humans may continue computing them while processing outcomes and actions (for instance, tracking them as metacognitive landmarks) but act in accordance with the MIX algorithm and switch to EU (or subjective values) only when the reliability of the latter is high enough (suggesting a dynamic switching between computational models (Steyvers et al., 2009; Rushworth et al., 2012; Wan Lee et al., 2014; O’Doherty et al., 2021). Both cases restrain us from the direct use of behavioral measures inherent to the expected utility or subjective valuation frameworks. Instead, risk-taking, loss-aversion, and other measures should be defined in terms of parameters of the MIX parameters which, in turn, is an essential research problem in light of the consistent elicitation of the additive strategy.

### Further Development of the MIX Model

In the current experimental protocol of the MIX task, the PRL task modified into an uncertain and volatile environment is employed, where reward frequencies of options are anti-correlated and stable throughout the experiment, and only the mapping between options and reward frequencies changes episodically. Modifying the MIX task into a more complex decision-making environment mimicking real-life situations and advancing the MIX algorithm to fit the human adaptive computational inferences will essentially contribute to current efforts in search of adaptive behavior models in environments where optimality is computationally intractable and physiologically implausible (e.g., Drugowitsch et al., 2016; Bossaerts and Murawski, 2017; Kwisthout and van Rooij, 2020).

The availability of only two options in the MIX task is an essential simplification of a decision-making environment, moreover, it may motivate the use of the heuristic of the additive strategy to take advantage of simple comparisons and learning of one option based on feedbacks of choosing the other option (Donahue and Lee, 2015). The MIX algorithm can trivially be generalized to a PRL task with more than two options. However, humans (and monkeys) may engage in sophisticated strategies of exploration and choice making (Daw et al., 2006; Payzan-LeNestour and Bossaerts, 2011; Gershman, 2019), thus challenging the MIX algorithm. Therefore, an empirical study with, for instance, three choices in the MIX task would test the applicability of the MIX algorithm or underlie its further development. Other generalization issues may arise if reward probabilities of relatively high- and low-ranked options are set to vary throughout the experiment. The strength of preferences for options may become volatile and not necessarily follow the true ranking (Gans et al., 2007; Yu and Cohen, 2009). Similarly, sensitivity to variations in proposed rewards of options are worth considering (Lauriola and Levin, 2001). Another critical research question is how the MIX model and its whole computational algorithm can be extended to environments with continuous rewards. Here, the generalization problem is the problem of binning rewards given computational and memory limitations of humans, especially in case of variations in environmental contingencies. So, endowing the experimental decision-making environment with features effectively mimicking real-life situations, engender new adaptive behaviors in subjects and advance the search for neurocomputational algorithms.

## Summary

The findings by Rouault et al. (2019) and Farashahi et al. (2019) have substantially extended behavioral and neural evidence that monkeys and humans employ an additive strategy of weighting reward magnitude and reward probability information into a decision variable in uncertain and volatile environments. Moreover, Rouault et al. (2019) have developed a learning and action selection algorithm by integrating distinct aspects of agent–environment interactions; confirmed it as a general model of value-based decision making encompassing the optimal model as a special case; and rejected the subjective value model, importantly, supporting the conclusions with both behavioral and neural evidence. The computational algorithm of the MIX model renders an analytical tool for studying the value-based decision making and modeling its underlying neural mechanisms in the prefrontal cortex. Further studies are needed to test and refine the MIX model in effectively accommodating behavioral variations and explaining corresponding neural activations in response to variations in the decision-making environment. Finally, gradually relaxing simplifications of the MIX task will allow us to generalize the MIX model to complex real-life decision-making environments.

## Data Availability Statement

The original contributions presented in the study are included in the article/supplementary material, further inquiries can be directed to the corresponding author.

## Author Contributions

AG drafted the manuscript. BG, VK, and EK edited and commented on the manuscript. All authors contributed to the article and approved the submitted version.

## Conflict of Interest

The authors declare that the research was conducted in the absence of any commercial or financial relationships that could be construed as a potential conflict of interest.

## Publisher’s Note

All claims expressed in this article are solely those of the authors and do not necessarily represent those of their affiliated organizations, or those of the publisher, the editors and the reviewers. Any product that may be evaluated in this article, or claim that may be made by its manufacturer, is not guaranteed or endorsed by the publisher.

## Footnotes

1 The encoding of computational components (state beliefs and normalized utilities) and their constituents (the reinforcement learning values and proposed rewards) are also described by Rouault et al. (2019)

2 In uncertainty, subjects do not possess explicit information about reward probabilities, hence, they are guided by beliefs, estimates of reward probabilities of options.
